# Microsatellite instability and ploidy status define three categories with distinctive prognostic impact in endometrioid endometrial cancer

**DOI:** 10.18632/oncotarget.2187

**Published:** 2014-07-08

**Authors:** Cristina Bilbao-Sieyro, Raquel Ramírez-Moreno, Germán Rodríguez-González, Orlando Falcón, Laureano León, Santiago Torres, Leandro Fernández, Sergio Alonso, Nicolás Díaz-Chico, Manuel Perucho, Juan Carlos Díaz-Chico

**Affiliations:** ^1^ Cancer Research Institute of The Canary Islands (ICIC), Las Palmas de Gran Canaria, Canary Islands, Spain; ^2^ Biochemistry, Molecular Biology and Physiology Department, Molecular and Translational Endocrinology Group, Institute for Biomedical and Health Research, Faculty of Health Sciences, Universidad de Las Palmas de Gran Canaria, Las Palmas de Gran Canaria, Canary Islands, Spain; ^3^ Obstetrics and Gynecology Department, Hospital Universitario Materno-Insular de Canarias, Las Palmas de Gran Canaria, Canary Islands, Spain; ^4^ Pathology Department, Hospital Universitario Materno-Insular de Canarias, Las Palmas de Gran Canaria, Canary Islands, Spain; ^5^ Clinical Sciences Department, Molecular and Translational Endocrinology Group, Institute for Biomedical and Health Research, Universidad de Las Palmas de Gran Canaria, Las Palmas de Gran Canaria, Canary Islands, Spain; ^6^ Institute of Predictive and Personalized Medicine of Cancer (IMPPC), Barcelona, Spain; ^7^ Sanford-Burnham Medical Research Institute (SBMRI), La Jolla, CA, USA; ^8^ Instituciò Catalana de Recerca i Estudis Avançats (ICREA), Passeig Lluis Companys 23, Barcelona, Spain

**Keywords:** endometroid, endometrial cancer, microsatellite instability, aneuploidy, survival

## Abstract

Microsatellite instability (MSI) and aneuploidy are inversely related phenomena. We tested whether ploidy status influences the clinical impact of MSI in endometrioid endometrial cancer (EEC). We analyzed 167 EECs for MSI and ploidy. Tumors were classified in three categories according to MSI and ploidy status. Associations with clinicopathological and molecular variables, survival, and treatment response were assessed.

All MSI tumors (23%) were scored as diploid, and 14% of microsatellite stable (MSS) tumors presented aneuploidy. MSI tumors associated with older age at diagnosis, non-obesity, high histological grade, and advanced surgical stage. MSS-aneuploid tumors also associated with higher grade and advanced stage. In multivariate survival analysis MSI did not influence disease-free survival (DFS) or cancer-specific survival (CSS). However, when just diploid tumors were considered for the analysis, MSI significantly contributed to worse DFS and CSS, and the same was observed for aneuploidy when MSS tumors were analyzed alone. In diploid tumors, a differential response to postoperative radiotherapy (RT) was observed according to MSI, since it predicted poor DFS and CSS in the multivariate analysis.

We conclude that ploidy status influences the clinical impact of MSI in EEC. Among diploid tumors those with MSI have poor clinical outcome and respond worse to RT.

## INTRODUCTION

Endometrial cancer (EC) is the most common pelvic gynecological malignancy and the fourth leading cause of female cancer in occidental countries [1]. Although the majority of cases follows an indolent course and are cured by surgery alone, about 20% of patients recur and die by disease despite receiving adjuvant therapies [1]. Two mayor types of EC are distinguished based on epidemiology, histopathology and clinical behavior. Type I endometrioid EC, which is the most abundant (about 80%), arises in relatively younger women with a history of unopposed estrogenic stimulation, usually has differentiated or moderately differentiated histological grade with non-aggressive behavior. Type II non endometrioid EC (mostly papillary serous and clear cell), commonly occurs in older women with a background unrelated to unopposed estrogen exposure, usually is high grade and has worse prognosis [2, 3]. The endometrioid type frequently presents microsatellite instability (MSI), estrogen (ER) and progesterone (PR) receptors expression, *PTEN*, *K-ras* and *β-Catenin* mutations, epigenetic silencing of *APC* and a near diploid DNA content, although aneuploidy has also been detected in around 20% of this type [4]. In contrast, the non endometrioid type is mostly aneuploid, with lack or weak ER and PR expression, *p53* mutations and HER2 overexpression [2, 3].

MSI is present in the majority of tumors of the hereditary nonpolyposis colorectal cancer (HNPCC) syndrome, and also in a subset (15-20%) of sporadic tumors [5-8], that accumulate hundreds of thousands of somatic clonal mutations in simple repeat sequences (microsatellites) as a result of a defective mismatch repair (MMR) system [6, 9]. In colorectal cancer (CRC) MSI tumors exhibit pseudodiploidy and better outcome compared to tumors without MSI (MSS), which frequently are aneuploid [6, 9, 10]. In CRC, loss of expression of DNA double strand breaks repair proteins has been associated to increased aneuploidy and poorer survival [11-14]. Accordingly, three different pathways based on the combination of these two anomalies, namely, MSI-diploid, MSS-diploid and MSS-aneuploid, have been proposed to better stratify CRC according to clinical characteristics and outcome [15-17].

In EC MSI is mainly present in near-diploid tumors of endometrioid histology [3], and shows different gene mutation profile compared to gastrointestinal tumors of the mutator phenotype [18-20], associating with *K-ras* and *PTEN* mutations [3]. Published data about the clinical impact of MSI are conflicting [21-26], and recent studies have related aneuploidy to worse clinical behavior [4, 27, 28].

In this study, we tested whether MSI and aneuploidy could interfere when determining its influence on clinicopathological characteristics and outcome in endometrioid EC based on the inverse relationship found between MSI and aneuploidy, and the precedents about the clinical effect of the combination of these two genomic instability phenotypes in CRC.

## RESULTS

In our series of 167 patients with endometrioid EC, all tumors with MSI presented *MLH1* promoter methylation and had a DNA-quasy-diploid content, and all aneuploid tumors were MSS. A single patient with presence of MSI and aneuploidy in the same tumor sample was excluded to simplify categories. Accordingly, tumors were divided in three categories: MSI, MSS-diploid and MSS-aneuploid. MSI and aneuploidy were detected in 33 (20%) and 24 (14%) cases respectively. The distribution of the subtypes according to demographic, surgicopathological and molecular variables of patients is shown in Table 1.

**Table 1:** MSI and ploidy status & demographic, surgico-pathological, and molecular characteristics of endometrioid endometrial cancer (EEC)

		MSI-diploid	MSS-diploid	MSS-aneuploid		MSI vs MSS
Characteristic	n	(n=33)^a^	(n=110)^a^	(n=24)^a^	p-value	p-value*
Age at diagnosis (years)^1^	167	67.3 ± 9.9	62.5 ± 9.4	62.2 ± 11.8	**0.040**	**0.035**
BMI (kg/m^2^)						
<30	66	21 (32) (64)^b^	37 (56) (34)^b^	8 (12) (33)^b^	**0.007**	**0.032**
>30	101	12 (12) (36)	73 (72) (66)	16 (16) (67)		
Diabetes						
no	125	22 (18)	84 (67)	19 (15)	>0.1	
yes	42	11 (26)	26 (62)	5 (12)		
Hypertension						
no	84	17 (20)	53 (63)	14 (17)	>0.1	
yes	83	16 (19)	57 (69)	10 (12)		
Age of menarche (years)^2^	167	13 (11-19)	13 (9-18)	13 (10-17)	>0.1	
No. of births						
'0-1	45	15 (33) (45)^b^	25 (56) (23)^b^	5 (11) (21)^b^	**0.016**	**0.023**
'2-3	52	9 (17) (27)	34 (65) (31)	9 (17) (38)		
'>3	70	9 (13) (27)	51(73) (46)	10 (14) (42)		
Total pregnancy months^2^^,^^3^	167	18 (0-81)	27 (0-126)	27 (0-90)	0.085	ns
Menopause						
no	17	2 (17)	10 (59)	5 (33)	>0.1	
yes	150	31 (24)	100 (67)	19 (16)		
Age of menopause (years)^2^	150	52 (40-57)	51 (30-60)	48 (44-55)	**0.043**	ns
Years of menstruation^1^^,^^4^	167	34.6 ± 5.5	32.2 ± 6.2	31.6 ± 4.9	0.087	ns
Years from menopause to diagnosis^1^	167	16.6 ± 10.7	13.1 ± 8.9	14.1 ± 11.1	>0.1	
Stage						
I	115	16 (14) (16)^c^	86 (75)	13 (11) (13)^d^	**0.001**	
II	27	8 (30) (30)	16 (59)	3 (11) (16)		
III	25	9 (36) (53)	8 (32)	8 (32) (50)		
Grade						
'1	88	14 (16) (17)^c^	68 (77)	6 (7) (8)^d^	**0.005**	
'2	51	10 (20) (25)	30 (59)	11 (22) (27)		
'3	28	9 (32) (43)	12 (43)	7 (25) (37)		
Myometrial infiltration						
0-<50%	121	25 (21)	81 (67)	15 (12)	ns	
>50%	46	8 (17)	29 (63)	9 (20)		
Vascular invasion						
no	122	18 (15) (16)^c^	92 (75)	12 (10) (12)^d^	**0.000**	
yes	45	15 (33) (45)	18 (40)	12 (27) (40)		
Treatment						
surgery	77	14 (18)	54 (70)	9 (12)	ns	
surgery+radiotherapy	90	19 (21)	56 (62)	15 (17)		
ER (fmol/mg prot.)^2^	167	58 (0-280)	95 (0-737)	48 (0-376)	**0.032**	
PgR (fmol/mg prot.)^2^	167	223 (0-1684)	330 (0-2069)	157 (0-1724)	ns	
S-phase (percentaje)^2^	167	7.7 (0.4-24.0)	4.3 (0.7-21.4)	7.0 (1.2-24.7)	**0.000**	
K-ras						
wild-type	143	20 (14)	102 (71)	21 (15)	**0.000**	
mutant	24	13 (54)	8 (33)	3 (13)		
PTEN						
wild-type	108	15 (14)	73 (68)	20 (19)	**0.010**	
mutant	59	18 (31)	37 (63)	4 (7)		
β-catenin						
wild-type	133	28 (21)	86 (65)	19 (14)	ns	
mutant	34	5 (15)	24 (71)	5 (15)		
APC						
unmethylated	109	10 (9)	84 (77)	15 (14)	**0.000**	
methylated	55	23 (42)	24 (44)	8 (15)		

* from binary logistic regression by comparing the MSI and MSS groups

a n (%), unless otherwise specified

b % in the specified category

c % in diploid group

d % in MSS group

1 mean ± SD

2 median (range)

3 cases with uncomplete pregnancy were unknown

4 calculated as age of menopause or age at diagnoses for premenopausal women - age of menarche - number of births * 1.125 ns = non significant

p=0.096

ER=estrogen receptor; PgR=progesterone receptor

p-values below 0.05 are in bold type

MSI tumors exhibited distinctive demographic features compared to MSS-aneuploid and MSS-diploid both of which shared similar features. Compared to MSS patients, those with MSI were older, relatively slim and had fewer births, in the multivariate analysis. Regarding clinico-pathological features, MSI and MSS-aneuploid tumors behaved similarly and exhibited marked differences compared to the MSS-diploid category. Thus, MSI and MSS-aneuploid tumors associated with advanced stage of progression (p=0.001) undifferentiated histological grade (p=0.005), and invasion of vascular space (p<0.001). With respect to molecular parameters, MSI tumors had higher frequency of *K-ras* (p<0.001) and *PTEN* (p=0.01) mutations, and *APC* promoter methylation (p<0.001). MSI and MSS-aneuploid categories associated with high S-phase (p<0.001).

**Figure 1:**
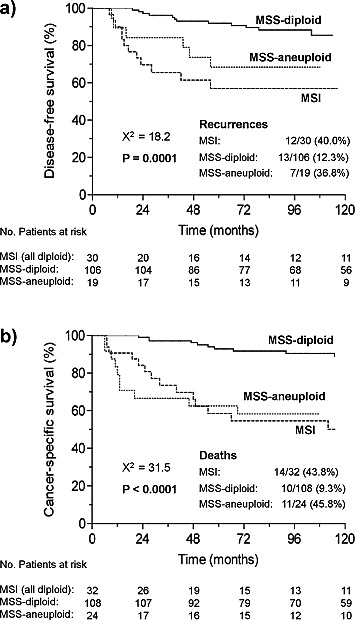
Kaplan-Meier curves for a) disease free survival and b) cancer-specific survival of the three categories of tumors defined by microsatellite instability and ploidy status in the whole series. Number of patients at risk, i.e. disease-free (a) or surviving (b), at time-points 0, 24, 48, 72, 96 and 120 months, are shown below the x-axis of each graph.

Univariate survival analysis (summary in Supplementary information, Tables S1 and S2) of the whole series showed that MSI, aneuploidy, older age at diagnosis, advanced stage, higher grade, myometrial invasion, vascular invasion, and radiation therapy, among others, were associated with worse DFS and CSS. Survival curves of the whole series stratified according to the three defined categories showed significant better DFS and CSS for MSS-diploid tumors (Fig. 1). The potential of MSI and aneuploidy as prognostic markers was estimated by multivariate analyses in the whole series and in the diploid and MSS groups (Tables 2 and 3). In the whole series, the basic multivariate DFS and CSS models included age at diagnosis, surgical stage and histological grade. After a stepwise procedure, MSI did not show significant prognostic value for DFS or CSS, and aneuploidy did for CSS (p=0.01).

**Table 2:** Multivariate disease-free survival analysis of EEC according to MSI and ploidy

	Whole series^1^			Diploid tumors			MSS tumors			Whole series^2^		
	(No. of patients = 155)			(No. of patients = 136)			(No. of patients = 125)			(No. of patients = 155)		
	(No. of events = 32)			(No. of events = 25)			(No. of events = 20)			(No. of events = 32)		
Characteristic	HR (95%CI)	Wald Χ^2^	p-value	HR (95%CI)	Wald Χ^2^	p-value	HR (95%CI)	Wald Χ^2^	p-value	HR (95%CI)	Wald Χ^2^	p-value
Age (years)	1.0 (1.0-1.1)	4.1	0.04	1.1 (1.0-1.1)	4.4	0.04			-	1.0 (1.0-1.1)	2.8	0.09
Stage			0.00			0.02			0.00			0.00
I	1*			1*			1*			1*		
II	2.5 (1.0-6.2)	4.0	0.05	3.6 (1.3-10.1)	5.7	0.02			0.72	2.7 (1.1-6.7)	4.4	0.04
III	8.2 (3.5-19.0)	23.8	0.00	6.9 (2.1-22.3)	10.4	0.01	11.8 (4.2-32.8)	22.4	0.00	5.6 (2.3-13.8)	14.3	0.00
Grade			0.03			0.23			0.03		5.5	0.06
'1	1*						1*			1*		
'2			0.15						0.15			0.40
'3	3.3 (1.4-8.2)	6.9	0.01				4.7 (1.4-15.2)	6.5	0.01	2.9 (1.2-7.2)	5.4	0.02
MSI status							NOT APPLICABLE			
MSS				1*						1*		
MSI			0,15	2.9 (1.2-6.9)	5.6	0.02				2.4 (1.0-5.9)	3.6	0.05
Ploidy				NOT APPLICABLE						
Diploid										1*		
Aneuploid			0,29						0,37	2.4 (1.0-6.4)	2.8	0.09

1 MS status and ploidy were added separately to the basic model

2 MS status and ploidy were added at the same time to the basic model

* Reference category

HR (95%CI) = hazard ratio (95% confidence interval)

**Table 3:** Multivariate cancer-specific survival analysis of EEC according to MSI and ploidy

	Whole series^1^			Diploid tumors			MSS tumors			Whole series^2^		
	(No. of patients = 164)			(No. of patients = 140)			(No. of patients = 132)			(No. of patients = 164)		
	(No. of events = 35)			(No. of events = 24)			(No. of events = 21)			(No. of events = 35)		
Characteristic	HR (95%CI)	Wald Χ^2^	p-value	HR (95%CI)	Wald Χ^2^	p-value	HR (95%CI)	Wald Χ^2^	p-value	HR (95%CI)	Wald Χ^2^	p-value
Age (years)	1.0 (1.0-1.1)	5.9	0.02	1.1 (1.0-1.1)	3.8	0.05	1.1 (1.0-1.1)	3.5	0.06	1.0 (1.0-1.1)	5.5	0.02
Stage			0.00			0.00			0.00			0.00
I	1*			1*			1*			1*		
II	3.3 (1.3-8.3)	6.7	0.01	3.6 (1.3-10.1)	5.7	0.02			0.39	3.8 (1.5-9.6)	7.7	0.00
III	10.8 (4.7-24.8)	31.4	0.00	6.9 (2.1-22.3)	10.4	0.00	11.1 (3.9-31.1)	20.8	0.00	7.5 (3.2-17.8)	20.8	0.00
Grade			0.00			0.02			0.00			0.00
'1	1*			1*			1*			1*		
'2	3.2 (1.3-8.3)	5.9	0.02	2.7 (0.9-8.2)	3.3	0.07	3.4 (1.0-11.4)	3.8	0.05	2.5 (1.0-6.4)	3.5	0.06
'3	7.1 (2.8-17.9)	16.8	0.00	4.7 (1.6-14.0)	7.9	0.00	8.8 (2.4-31.7)	11.1	0.00	6.3 (2.5-16.1)	14.7	0.01
MSI status							NOT APPLICABLE			
MSS	1*			1*						1*		
MSI			0.36	3.0 (1.2-7.5)	5.5	0.02				2.5 (1.0-6.2)	3.9	>0.05
Ploidy				NOT APPLICABLE						
Diploid	1*									1*		
Aneuploid	2.7 (1.2-5.8)	6.2	0.01						0,37	4.1 (1.6-11.2)	9.0	0.00

1 MS status and ploidy were added separately to the basic model

2 MS status and ploidy were added at the same time to the basic model

* Reference category

HR (95%CI) = hazard ratio (95% confidence interval)

In the DNA-diploid group, age, grade and stage were also present in the basic DFS and CSS models. When MSI was tested, it significantly associated with poor DFS (p=0.02) and CSS (p=0.02). In the MSS group, stage and grade for DFS, and age, stage and grade for CSS, were included in the basic model. Aneuploidy was not associated with DFS, but remained significant for CSS (p=0.02).

The significant prognostic value of MSI in the DNA-diploid group, and of aneuploidy in the MSS group, prompted us to estimate the impact of adding both variables simultaneously to the basic model in the whole series. As a result, MSI was significantly associated with poor DFS (p=0.05), and MSI and aneuploidy with worse CSS (p<0.05 and p<0.01, respectively). Thus, patients with MSI had an increased risk of recurrence and die by disease 2.4 (95% CI, 1.0 to 5.9) and 2.5 times (95% CI, 1.0-6.2) respectively, compared to MSS-diploid tumors.

**Figure 2:**
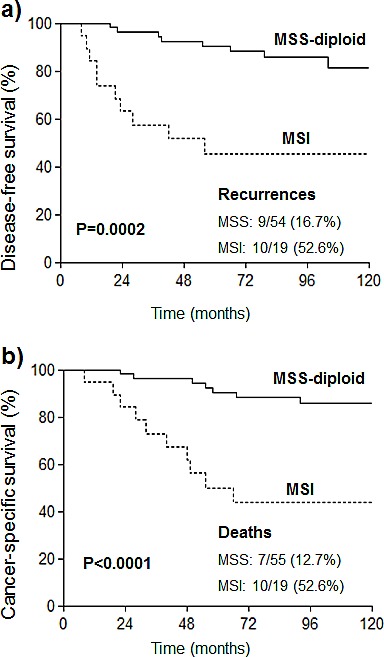
Kaplan-Meier curves for a) disease free survival and b) cancer-specific survival of EC patients with diploid tumors (MSS or MSI) who were treated with radiotherapy

The clinical impact of MSI was also assessed among the subgroup of patients treated with postoperative radiotherapy (RT) (Figure 2, univariate analyses are summarized in Supplementary information, Table S3). In the whole series of these treated patients, age, stage and grade, and in the DNA-diploid group, age and stage, were incorporated in the basic model for DFS and CSS. According to the multivariate analysis, MSI lacked independent value for DFS and CSS in the whole series. However, in the group of diploid tumors, MSI predicted worse DFS (p=0.02) and CSS (p=0.04) (Table 4).

**Table 4:** Multivariate survival analysis for MSI after treatment with radiotherapy

	Disease-free survival						Cancer-specific survival					
	Whole series			Diploid tumors			Whole series			Diploid tumors		
	(No. of patients = 85)			(No. of patients = 73)			(No. of patients = 89)			(No. of patients = 74)		
	(No. of events = 25)			(No. of events = 19)			(No. of events = 25)			(No. of events = 17)		
Characteristic	HR (95%CI)	Wald Χ^2^	p-value	HR (95%CI)	Wald Χ^2^	p-value	HR (95%CI)	Wald Χ^2^	p-value	HR (95%CI)	Wald Χ^2^	p-value
Age (years)	1.0 (1.0-1.1)	3.0	0.08	1.1 (1.0-1.1)	3.7	0.06	1.1 (1.0-1.1)	6.0	0.01	1.1 (1.0-1.1)	4.6	0.03
Stage			0.03			0.30			0.00			0.07
I	1*						1*			1*		
II			0.17				2.6 (0.9-7.8)	3.0	0.08	4.1 (1.1-14.9)	4.7	0.03
III	4.5 (1.5-13.3)	7.1	0.01				7.5 (2.5-22.9)	12.5	0.00	4.1 (0.9-20.0)	3.2	0.07
Grade			0.16			-			0.06			-
'1							1*					
'2									0.21			
'3							3.9 (1.3-11.7)	5.7	0.02			
MS status												
MSS				1*						1*		
MSI			0,23	3.6 (1.2-10.7)	5.4	0.02			0.73	3.4 (1.1-11.1)	4.2	0.04

* Reference category

HR (95%CI) = hazard ratio (95% confidence interval)

## DISCUSSION

In EC MSI and aneuploidy appear as mutually exclusive genomic alterations with clinical implications, although clinical data regarding MMR deficiency remain conflicting. In this work we hypothesized whether the clinical relevance of MSI in endometrioid EC could be clarified by studying this genetic alteration under the perspective of three alternative genomic cancer pathways, namely, MSI, MSS-diploid, and MSS-aneuploid. The clear known differences between endometrioid and non-endometrioid tumors [2, 3] justify the exclusion of non-endometrioid tumors from our study, since they can act as a confounding factor [26]. Another circumstance that added homogeneity to the stratification of our series is that all MSI tumors were diploid and had *MLH1* promoter methylation. This refers to a non-hereditary origin [29], especially relevant when studying the relationship between MSI and, at least, some demographic variables.

Patient age, obesity and nulliparity are three known EC risk factors. Although previous studies failed to find any relation between MSI and age [22, 24], it has been shown that women displaying MSI and methylated *MLH1* are significantly older [30, 26]. This agrees with our observations and with the fact that *MLH1* methylation is known to be an age related phenomena [31-33]. The inverse relationship between MSI and BMI is in accordance to other reports [30]. In CRC the association between MSI and nulliparity has also been published [32]. Although a connection between estrogen exposure and reduced risk of acquiring MMR deficiency has been suggested [32, 34], further studies are required to elucidate the molecular bases.

In the whole series, MSI cases associated with higher FIGO grade, advanced surgical stage and lymphovascular invasion compared to MSS-diploid tumors. For each clinical feature, several groups have reported similar results, either in EC, without distinction of histological types, or in the endometrioid subtype [24, 26], although discordant findings have also been published [22, 24, 30, 26]. In CRC, the association between MSI and poor differentiated grade is well established [10]. Our data show that MSI might manifest in clones with undifferentiated aggressive appearing histology among tumors with a background of DNA-diploid content. MSS-aneuploid subtype tumors had pathological features similar to those of MSI tumors, since they frequently presented higher grade, advanced stage and vascular involvement. The association of aneuploidy with a more aggressive clinical phenotype in EC is well documented [4, 27, 28]. According to our data, MSS-diploid tumors display a more benign phenotype (differentiated grade, early stage and absence of vascular invasion) compared to MSI and MSS-aneuploid tumors.

We found that MSI tumors were more likely to present lower ER levels and a trend towards lower PgR, which contrast with the fact that endometrioid tumors are prototypically associated with estrogen stimulation and generally express both receptors [2]. The reason for this observation could be the high prevalence of MSI in poor differentiated grades, since most high grade EC show weak ER and PgR expression [2]. In CRC, either arising in Lynch syndrome families or not, significantly lower hormone receptor levels have been described in MSI tumors [35, 36].

Our EC MSI tumors also showed increased S-phase compared to MSS-diploid tumors. In this line, cyclin A, which is necessary for DNA replication and S-phase entry, has also been related to the MSI phenotype and to cell proliferation in EC [37, 38].

As previously reported [39, 40, 3], our results show that MSI was positively related to *K-ras* and *PTEN* mutations and *APC* promoter hypermethylation, whereas inversely associated with *β-catenin* mutations. It is well known that in CRC the association of MSI with these molecular variables is quite different. For example, *K-ras* mutations mainly coexist with MSS tumors, while *β-catenin* mutations and *APC* promoter methylation are characteristic of MSI tumors [6, 41, 42].

The absence of a significant impact of MSI on survival was recently reported by Zighelboim et al [26] and by others previously in endometrioid EC, and by Basil et al [21] and McDonnald et al [22], among others, in EC (endometrioid and non-endometrioid). Conversely, several groups have referred an association of MSI with better [23, 24], or worse [25] outcome in series including only endometrioid tumors or both histological types. As for the prognostic value of ploidy in EC, latest publications suggest that is a statistically independent prognosticator of poor outcome through multivariate analysis [4, 27, 28].

Our most relevant finding in the multivariate survival analysis was that both MSI and aneuploidy remained as significant prognosticators of worse DFS and CSS when aneuploidy and MSI where, respectively, excluded from the corresponding survival analyses. In the whole series, the simultaneous inclusion of both variables influenced their respective outcome values, making (marginally) significant its relationship with DFS, and significant its contribution to CSS. This suggests that when analysing the prognostic value of MSI or aneuploidy, each variable could act as a confounding factor for the other. It is noteworthy that the number of recurrences (40%) and death (44%) of patients with MSI EC tumors in our series was higher than previously reported by some studies [22-24, 26]. In CRC, the interference between these variables in clinical outcome has also been proposed, and cumulative evidence associates MSI-diploidy with good prognosis [16, 17]. The strong local and systemic antitumoral immune response observed in MSI CRC have been proposed as a possible mechanism to explain the increased survival rates in patients with MSI tumors [43]. However, in endometrioid EC, Risinger et al [44] reported that significant differences in tumor infiltrating lymphocytes derived transcripts, do not occur between tumors with and without MSI. Therefore, a relevant task for future research will be to elucidate the mechanism beneath the different immune response of CRC and EC with MSI, and why the influence of MSI-diploidy on patient's survival in EC is just the opposite of that observed in CRC.

Finally, we have also observed that among diploid tumors, MSI predicts worse DFS and CSS in patients treated with postoperative RT. We have previously suggested the predictive role of MSI in early staged radiation-treated endometrioid EC [45]. There are few related studies, mostly in preoperative RT rectal treated tumors, and with discordant results [46-48]. As previously pointed, the worse response of MSI tumors to RT could be related to the suggested increased malignant potential of *MLH1* null mice after ionizing radiation exposure due to the activation of mitotic recombinational events [49].

Due to the combination of low incidence of MSI and aneuploidy and of recurrences and cancer-specific deaths inherent to this non-aggressive endometrial cancer type, the present work has limited statistical power. Nevertheless, there was sufficient data (at least 5 events for each explanatory variable) to get reproducible results from multivariate regression analysis. Future work thus seems warranted, as it may assist to a better stratification of patients for election of modality of treatment. In summary, our data show that the three groups described here based on MSI status and ploidy might represent different molecular pathways of tumorigenesis and progression of endometrioid endometrial cancer with different prognostic and predictive significance.

## MATERIALS AND METHODS

### Study Participants and Clinical Data

A series of 167 patients with endometrioid EC (surgical stage I-III), was selected from a previously described Caucasian population-based series of 204 patients with EC, diagnosed and treated between 1990 and 1999 [40]. Exclusion causes from the original EC series were: non endometrioid histology, fulfill the Amsterdam criteria of HNPCC and lack of fresh tumor sample to perform flow cytometry. A single patient with presence of MSI and aneuploidy in the same tumor sample was also excluded.

Clinico-pathological data of patients were prospectively collected and computerized. Surgical stage and tumor grade were assigned according to the International Federation of Gynecology and Obstetrics (FIGO) 1988 criteria. None of the patients received preoperative radiation or chemotherapy. All patients underwent exploratory laparatomy, total hysterectomy and bilateral salpingo-oophorectomy. Lymphadenectomy was performed at the discretion of the surgeon. Radiation therapy after surgery was administered to most of stage II and III patients and to stage I patients with risk factors as deep myometrial invasion and high histological grade. The treatment consisted of irradiation of either the vaginal cuff (45-60 Gy), the whole pelvis (50 Gy), or both. A few patients received irradiation in the whole pelvis and in the para-aortic nodes (45 Gy). Only 4 patients received adjuvant chemotherapy in addition to radiotherapy. Forty non-cancerous endometrial samples were used as control tissue in methylation analysis. The study was approved by the institutional ethical committee and all subjects provided informed consent.

### Tissue fractionation, DNA isolation and molecular analyses

Frozen tumor specimens were macroscopically dissected to enrich for neoplastic cellularity. The tissue was mechanically disrupted and the sub-cellular fractions (cytosolic and nuclear) were obtained as previously described [50]. DNA was extracted by a standard procedure. DNA was also obtained from matching peripheral blood leukocytes and from non-tumoral endometrial tissues.

For MSI classification, five quasi monomorphic mononucleotide markers BAT25, BAT26, NR21, NR24 and NR27 were used according to published conditions and criteria [51, 45]. Tumors were considered MSI when they showed alterations in at least 2 out of five markers. Ninety seven percent of MSI tumors (all but one) were correctly classified by using only BAT-26 and BAT-25. Tumors with only one altered marker (n=12) were classified as MSS [52].

Promoter methylation analysis of *hMLH1* gene was performed by methylation-specific PCR (MSP) [53], using previously reported primers and conditions [42, 54]. Methylation of the analyzed *MLH1* promoter region invariably correlates with absence of gene expression [55].

Ploidy status and S-phase fraction were estimated by flow cytometry as previously reported [50]. Tumors with a DNA index > 0.9 and < 1.1 were classified as “(quasi)diploid” and others as aneuploid.

Single strand conformation polymorphism (SSCP) and DNA sequencing analyses were applied to search for mutations in K-*ras* (exons 1 and 2), *PTEN* (exons 1 to 9 and intronic splice sites) and *β-catenin* (*CTNNB1*) (exon 3 mutations and intragenic deletions). Analyses of *PTEN* and *β-catenin* were as described elsewhere [41, 40]. Primer sequences and conditions for *K-ras* mutation analysis are available upon request. Promoter methylation analysis of *APC* was performed by methylation-specific PCR (MSP) [53], using previously reported primers and conditions [42, 54]. Quantification of estrogen (ER) and progesterone (PR) receptors was done using the labeled hormone exchange method as described [50].

### Follow-up

All patients were routinely followed at 3-month intervals the first 2 years, 6 months until the fifth year, and every year thereafter. Three patients lost in less than 6 months after surgery were excluded. Disease-free survival (DFS) was defined as the time from surgery to recurrence for patients with a disease-free interval after surgery, and cancer-specific survival (CSS) as the time from surgery to cancer-related death. Patients with persistent disease after surgery (n=9) were excluded from DFS analysis. Patients that died by causes other than disease (n=10) were censored at the date of death. The median follow-up was 112 months (range 6-227 months) for all patients and 132 months (range 16-227 months) for patients that did not die by disease.

### Statistical analyses

The relationship among categories established by MSI and ploidy status and categorical variables was analyzed by Chi-square test. ANOVA was used for variables with a Gaussian distribution, and Kruskal-Wallis test for variables with a non-normal distribution. Binary logistic regression analysis was employed to test the independent contribution of demographic variables to MSI development. Survival curves were prepared according to Kaplan-Meier method and compared by the log–rank test. Univariate and multivariate survival analysis was carried out using the Cox proportional hazard model. Demographic and surgicopathological (including treatment) variables were entered to discern their independent contribution to the basic regression model for the multivariate survival analyses. A forward stepwise method was used to test the influence of explanatory variables (with a p-value < 0.1 in the univariate analysis) in the multivariate regression studies. The analysis of independence was performed by likelihood ratio tests. All analyses were two sided, and statistical significance was set at a p-value < 0.05. Analyses were carried out using the PASW (version 18.0) statistical package (SPSS, Chicago, IL).

## SUPPLEMENTARY MATERIAL AND TABLES


