# Supplementary material for: Microsatellite instability and ploidy status define three categories with distinctive prognostic impact in endometrioid endometrial cancer
Source: Oncotarget. 2014 Jul 8;5(15):6206–17. doi: 10.18632/oncotarget.2187 (PMC4171623; doi:10.18632/oncotarget.2187)
Supplement: Supplementary file 1 [file oncotarget-05-6206-s001.docx]

Microsatellite instability and ploidy status define three categories with distinctive prognostic impact in endometrioid endometrial cancer

Supplementary Material

**Supplementary Table S1: Univariate disease-free survival analysis for demographic and surgico- pathological variables, and for MSI and aneuploidy, in the whole series and subgroups of patients**

|  | | Whole series |  | Diploid tumors |  | MSS tumors |
| --- | --- | --- | --- | --- | --- | --- |
| Characteristic |  | p-value |  | p-value |  | p-value |
| Age at diagnosis (years) |  | **0.012** |  | **0.002** |  | >0.1 |
| BMI (kg/m2)  Diabetes |  | **0.040**  >0.1 |  | 0.054  >0.1 |  | >0.1  >0.1 |
| Hypertension |  | >0.1 |  | >0.1 |  | >0.1 |
| Age of menarche (years) |  | **0.040** |  | **0.049** |  | 0.089 |
| No. of births |  | >0.1 |  | >0.1 |  | >0.1 |
| Total pregnancy months |  | >0.1 |  | >0.1 |  | >0.1 |
| Menopause |  | >0.1 |  | 0.092 |  | >0.1 |
| Age of menopause (years) |  | >0.1 |  | >0.1 |  | >0.1 |
| Years of menstruation |  | >0.1 |  | >0.1 |  | >0.1 |
| Years from menopause to diagnosis |  | **0.007** |  | **0.003** |  | >0.1 |
| Stage |  | **0.000** |  | **0.000** |  | **0.000** |
| Grade |  | **0.002** |  | **0.023** |  | 0.079 |
| Myometrial infiltration |  | 0.083 |  | >0.1 |  | >0.1 |
| Vascular invasion |  | **0.000** |  | **0.012** |  | **0.007** |
| Treatment |  | **0.010** |  | **0.030** |  | >0.1 |
| MS status |  | **0.001** |  | **0.000** |  | - |
| Ploidy |  | 0.088 |  | - |  | **0.010** |

**Supplementary Table S2: MSI and aneuploidy in univariate cancer-specific survival analysis**

|  | | Whole series |  | Diploid tumors |  | MSS tumors |
| --- | --- | --- | --- | --- | --- | --- |
| Characteristic |  | p-value |  | p-value |  | p-value |
| Age at diagnosis (years) |  | **0.002** |  | **0.001** |  | **0.032** |
| BMI (kg/m2)  Diabetes |  | 0.060  >0.1 |  | 0.090  >0.1 |  | >0.1  >0.1 |
| Hypertension |  | >0.1 |  | >0.1 |  | >0.1 |
| Age of menarche (years) |  | >0.1 |  | >0.1 |  | >0.1 |
| No. of births |  | >0.1 |  | >0.1 |  | >0.1 |
| Total pregnancy months |  | >0.1 |  | >0.1 |  | >0.1 |
| Menopause |  | >0.1 |  | >0.1 |  | >0.1 |
| Age of menopause (years) |  | >0.1 |  | >0.1 |  | >0.1 |
| Years of menstruation |  | >0.1 |  | >0.1 |  | >0.1 |
| Years from menopause to diagnosis |  | **0.004** |  | **0.006** |  | >0.1 |
| Stage |  | **0.000** |  | **0.000** |  | **0.000** |
| Grade |  | **0.000** |  | **0.000** |  | **0.001** |
| Myometrial infiltration |  | **0.001** |  | 0.083 |  | **0.003** |
| Vascular invasion |  | **0.000** |  | **0.003** |  | **0.000** |
| Treatment |  | 0.052 |  | >0.1 |  | >0.1 |
| MSI status |  | **0.000** |  | **0.000** |  | - |
| Ploidy |  | **0.001** |  | - |  | **0.000** |

**Supplementary Table S3: Univariate disease-free and cancer-specific survival analysis for demographic and surgico-pathological variables, and for MSI, in patients treated with radiotherapy after surgery**

Disease-free survival

Cancer-specific survival

|  | | Whole series |  | Diploid tumors |  | Whole series |  | Diploid tumors |
| --- | --- | --- | --- | --- | --- | --- | --- | --- |
| Characteristic |  | p-value |  | p-value |  | p-value |  | p-value |
| Age at diagnosis (years) |  | **0.011** |  | **0.009** |  | **0.001** |  | **0.006** |
| BMI (kg/m2) |  | 0.072 |  | >0.1 |  | >0.1 |  | >0.1 |
| Diabetes |  | >0.1 |  | >0.1 |  | >0.1 |  | >0.1 |
| Hypertension |  | >0.1 |  | >0.1 |  | >0.1 |  | >0.1 |
| Age of menarche (years) |  | 0.051 |  | 0.072 |  | >0.1 |  | >0.1 |
| No. of births |  | **0.023** |  | >0.1 |  | >0.1 |  | 0.091 |
| Total pregnancy months |  | >0.1 |  | >0.1 |  | >0.1 |  | >0.1 |
| Menopause |  | >0.1 |  | >0.1 |  | >0.1 |  | >0.1 |
| Age of menopause (years) |  | >0.1 |  | >0.1 |  | >0.1 |  | >0.1 |
| Years of menstruation |  | >0.1 |  | >0.1 |  | >0.1 |  | >0.1 |
| Years from menopause to diagnosis |  | **0.024** |  | **0.024** |  | **0.007** |  | **0.014** |
| Stage |  | **0.001** |  | **0.015** |  | **0.000** |  | **0.002** |
| Grade |  | 0.070 |  | >0.1 |  | **0.021** |  | 0.071 |
| Myometrial infiltration |  | >0.1 |  | >0.1 |  | >0.1 |  | >0.1 |
| Vascular invasion |  | **0.036** |  | >0.1 |  | **0.002** |  | >0.1 |
| MS status |  | **0.003** |  | **0.001** |  | **0.006** |  | **0.000** |
